# Prioritisation by FIT to mitigate the impact of delays in the 2-week wait colorectal cancer referral pathway during the COVID-19 pandemic: a UK modelling study

**DOI:** 10.1136/gutjnl-2020-321650

**Published:** 2020-08-27

**Authors:** Chey Loveday, Amit Sud, Michael E Jones, John Broggio, Stephen Scott, Firza Gronthound, Beth Torr, Alice Garrett, David L Nicol, Shaman Jhanji, Stephen A Boyce, Matthew Williams, Claire Barry, Elio Riboli, Emma Kipps, Ethna McFerran, David C Muller, Georgios Lyratzopoulos, Mark Lawler, Muti Abulafi, Richard S Houlston, Clare Turnbull

**Affiliations:** 1 Division of Genetics and Epidemiology, Institute of Cancer Research, London, UK; 2 National Cancer Registration and Analysis Service, Public Health England, London, UK; 3 RM Partners, West London Cancer Alliance, London, UK; 4 Microbiology, Royal Marsden NHS Foundation Trust, London, UK; 5 Urology Unit, Royal Marsden NHS Foundation Trust, London, UK; 6 Division of Clinical Studies, Institute of Cancer Research, London, UK; 7 Department of Anaesthesia, Perioperative Medicine and Critical Care, Royal Marsden NHS Foundation Trust, London, UK; 8 Division of Cancer Biology, Institute of Cancer Research, London, UK; 9 Department of Colorectal Surgery, Oxford University Hospitals NHS Foundation Trust, Oxford, UK; 10 Department of Clinical Oncology, Imperial College Healthcare NHS Trust, London, UK; 11 Computational Oncology Group, Imperial College London, London, UK; 12 School of Public Health, Imperial College London, London, UK; 13 The Breast Unit, Royal Marsden NHS Foundation Trust, London, UK; 14 Patrick G Johnston Centre for Cancer Research, Queens University Belfast, Belfast, UK; 15 Epidemiology of Cancer Healthcare & Outcomes (ECHO) Group, University College London, London, UK; 16 Colorectal Surgery, Croydon Health Services NHS Trust, Croydon, London, UK; 17 Department of Clinical Genetics, Royal Marsden NHS Foundation Trust, London, UK

**Keywords:** colorectal cancer, colonoscopy, colorectal cancer screening

## Abstract

**Objective:**

To evaluate the impact of faecal immunochemical testing (FIT) prioritisation to mitigate the impact of delays in the colorectal cancer (CRC) urgent diagnostic (2-week-wait (2WW)) pathway consequent from the COVID-19 pandemic.

**Design:**

We modelled the reduction in CRC survival and life years lost resultant from per-patient delays of 2–6 months in the 2WW pathway. We stratified by age group, individual-level benefit in CRC survival versus age-specific nosocomial COVID-19–related fatality per referred patient undergoing colonoscopy. We modelled mitigation strategies using thresholds of FIT triage of 2, 10 and 150 µg Hb/g to prioritise 2WW referrals for colonoscopy. To construct the underlying models, we employed 10-year net CRC survival for England 2008–2017, 2WW pathway CRC case and referral volumes and per-day-delay HRs generated from observational studies of diagnosis-to-treatment interval.

**Results:**

Delay of 2/4/6 months across all 11 266 patients with CRC diagnosed per typical year via the 2WW pathway were estimated to result in 653/1419/2250 attributable deaths and loss of 9214/20 315/32 799 life years. Risk–benefit from urgent investigatory referral is particularly sensitive to nosocomial COVID-19 rates for patients aged >60. Prioritisation out of delay for the 18% of symptomatic referrals with FIT >10 µg Hb/g would avoid 89% of these deaths attributable to presentational/diagnostic delay while reducing immediate requirement for colonoscopy by >80%.

**Conclusions:**

Delays in the pathway to CRC diagnosis and treatment have potential to cause significant mortality and loss of life years. FIT triage of symptomatic patients in primary care could streamline access to colonoscopy, reduce delays for true-positive CRC cases and reduce nosocomial COVID-19 mortality in older true-negative 2WW referrals. However, this strategy offers benefit only in short-term rationalisation of limited endoscopy services: the appreciable false-negative rate of FIT in symptomatic patients means most colonoscopies will still be required.

**Significance of this study**

**What is already known on this subject?**

- COVID-19 has resulted in disruption across cancer diagnostics and surgery, affecting colorectal cancer (CRC) in particular through widespread shutdown of routine endoscopy due to safety concerns and under-staffing. Previous observational studies have quantified the impact on long-term survival of delay to treatment in CRC, although with inherent confounding by indication. Routinely generated data from Public Health England demonstrate varying proportions of CRCs presenting via urgent symptomatic, routine, screening and emergency routes. There has to date been no direct evaluation of the impact on survival of universal delays to diagnosis, in particular, focused on specific routes to diagnosis. Most data on faecal immunochemical testing (FIT) pertain to its role in the context of population screening, although some limited results are available from studies in symptomatic patients.

**What are the new findings?**

- We provide the first explicit modelling of FIT triage in the urgent symptomatic (2-week wait) population to mitigate survival decrement from delays of 2, 4 and 6 months, examining thresholds of 2, 10 and 150 µg Hb/g. We quantify impact through modelling of the impact on long-term (10-year) survival of variable delays in CRC diagnosis in symptomatic patients. We provide explicit modelling of the impact of different rates of nosocomial COVID-19 infection on the survival benefit for those referred for diagnostic colonoscopy.

**Significance of this study**

**How might it impact on clinical practice in the foreseeable future?**

- CRC is a common malignancy with high mortality. Delays in urgent referral of symptomatic patients and bottlenecks in endoscopy have potential to cause high attributable deaths and lost life years. FIT triage at 10 µg Hb/g offers opportunity to mitigate 89% of these deaths and reduce exposure of patients to nosocomial COVID-19 infection.

## Introduction

The COVID-19 pandemic has placed unprecedented pressure on healthcare services. Urgent redeployment of staff towards the management of COVID-19 cases within primary and secondary care has necessitated deprioritisation of non-COVID-19–related non-emergency clinical services, while elective surgery has been compromised by competition for anaesthetic and critical care staff. Non-COVID-19–related healthcare services may suffer years of disruption until the emergence of vaccines or effective treatment for COVID-19, in particular, if there are recurrent ‘waves’ of infection.

For many conditions, delay to treatment will impact quality of life but is unlikely to have long-term consequences. For patients with localised cancer, however, delay to diagnosis and treatment increase the likelihood of metastatic disease, with some tumours progressing from being curable by surgery (or radiotherapy), with near-normal life expectancy, to being incurable, with very limited life expectancy.^1^

Lockdown and public anxiety have each contributed to the significant reduction in presentation and referral from primary into secondary care of patients with symptoms of cancer.^2 3^ Reports of high rates of nosocomial COVID-19 infection and mortality present a dilemma to primary care physicians regarding the risk–benefit trade-off of investigatory referral into hospital for older patients with symptoms suggestive of cancer.^4^

Colorectal cancer (CRC) is the fourth most common cancer in the UK and second most common cause of cancer deaths.^5^ Survival for CRC in the UK has improved over the last 20 years with more patients diagnosed at an earlier stage and fewer emergency presentations, for which outcomes are typically poor.^6–8^ This is in part attributable to national CRC screening, which accounts for about 10% of diagnoses. Also important has been the establishment of the rapid access ‘2-week wait’ (2WW) urgent referral pathway for patients with specified ‘red-flag’ symptoms, through which 32% of CRCs are typically diagnosed^9 10^ (see online supplementary materials). Thirty-four per cent of CRC diagnoses are via ‘routine’ referral, which includes those under long-term surveillance for polyps, high-risk conditions and/or family history. Twenty-four per cent of CRCs are still diagnosed via emergency referral/presentation.

10.1136/gutjnl-2020-321650.supp1 Supplementary data

There is evidence of significant COVID-19–related disruption at multiple points in the CRC pathway to diagnosis and treatment. Recommendations from the British Society of Gastroenterology were issued in March 2020 indicating that, due to safety concerns, routine diagnostic endoscopy be suspended, causing disruption to all routes to diagnosis and suspension of the National Bowel Cancer Screening programme.^11–13^ Routine laparoscopic bowel resections were discontinued on account of risk of aerosol generation.^14^ Open bowel resections typically require some critical care unit (CCU) postoperative care, for which capacity has been under competition. In addition, there was widespread redeployment to the COVID-19 wards and CCU of acute general medical and surgical staff, including gastroenterologists and colorectal surgeons.

Faecal immunochemical testing (FIT) is widely used in CRC programmes of screening of asymptomatic individuals. FIT positivity currently is only used as an additional ‘rule-in’ criterion for urgent 2WW referral for individuals whose symptoms otherwise do not qualify.^9^ However, in some regions of the UK in response to capacity issues due to the COVID-19 pandemic, FIT has been implemented for systematic triage for colonoscopy of symptomatic 2WW patients. Recommendations released in March 2020 specified that all symptomatic patients evaluated in primary care should undergo FIT testing, with only those with FIT >10 µg Hb/g referred into secondary care and those with FIT >150 µg Hb/g prioritised for colonoscopy.^13 15 16^

**Figure 1:**
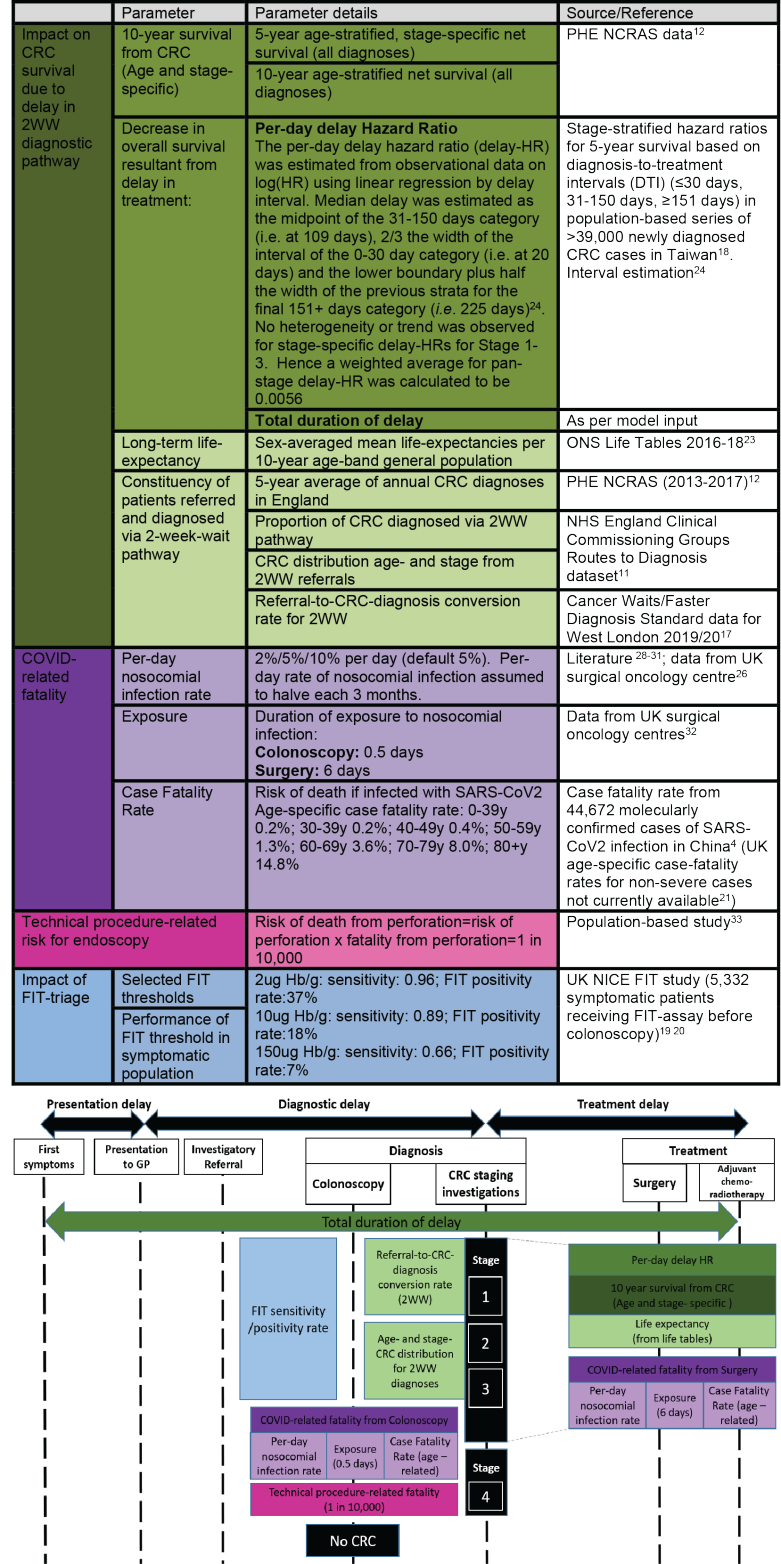
Description of model components and parameter estimates. See online supplementary materials for full model and online supplementary table 1 for sensitivity testing of parameters.

To inform clinical decision-making, we examined the impact on CRC survival of putative durations of pathway delay for symptomatic 2WW patients, accounting for competing survival impact from nosocomial COVID-19. We considered also the risk/benefit profile at different rates of nosocomial infection of investigatory referral for colonoscopy per referred patient (figure 1). We examined how prioritisation based on FIT thresholds could mitigate the impact of diagnostic delay, short-term pressures on endoscopy services and incidence of nosocomial infection.

10.1136/gutjnl-2020-321650.supp2 Supplementary data

## Methods

### Data sources

We obtained 5-year (2013–2017) and 10-year (2008–2017) net cancer survival data from Public Health England National Cancer Registration and Analysis Service (NCRAS)^6^ and life tables for 2016–2018 from UK Office of National Statistics (ONS).^16^ We obtained data on route to diagnosis with age and stage distribution from NHS England Clinical Commissioning Groups.^10^ For referral-to-CRC-diagnosis conversion rates, we used data from Cancer Waits/Faster Diagnosis Standard data for West London 2019/2020.^17^ For estimates of impact on survival of delays in the CRC pathway, we used published observational data on overall survival (OS) at 5 years by stage at diagnosis for >39 000 CRCs from a population-based registry in Taiwan, in which delayed diagnosis-to-treatment intervals (DTIs) were captured (≤30 days (standard), 31–150 days, ≥151 days).^18^ We obtained data on positive predictive value, negative predictive value, sensitivity, specificity and positivity for FIT at different thresholds from interim analysis of the UK NICE FIT study, in which 5332 symptomatic patients received a FIT assay before colonoscopy.^19 20^ Median duration of hospital stay was based on information from three large UK surgical oncology centres. We used data from Wuhan as the basis for mortality associated with COVID-19 (UK COVID-19 case-fatality rates are currently available only for hospitalised (severe) cases).^4 21^

### Analysis

#### Impact of COVID-associated delay on outcomes

We considered a 1-year overall period of disruption with per-patient pathway delays of 2, 4 or 6 months. We estimated per individual within each age-specific and stage-specific strata, 10-year net survival and life-years (LYs) gained from cancer treatment under ‘current’ (COVID-19) conditions and with specified durations of delay. For each age-specific and stage-specific stratum of CRCs diagnosed through the 2WW pathway, we estimated per year the attributable deaths and LYs lost from delay per stratum and in aggregate.

##### Ten-year net survival

Given statistical cure in patients with CRC is recognised to occur at 7–8 years post-diagnosis, we used 10-year stage-specific survival data in our analyses. Because 10-year stage-specific survival data for England are not available for recent cohorts, we estimated these by applying the ratio of stage-specific/all-stage from 5-year survival data (2013–2017) to most recently available 10-year all-stage data (2008–2017).^6 22^ Net 10-year survival estimates were used as a proxy for CRC-specific mortality, as background age-specific death rates have been adjusted to reflect cancer-specific mortality. To estimate average LYs gained per patient, we used the midpoint per 10-year age group for life expectancy (ONS).^23^

##### Per-day HR for delay in management

We extracted from observational data on DTI for >39 000 CRC cases the survival estimates for those with stage 1–3 disease, which captures the impact for those with potentially curable disease of within-stage progression, between-stage progression and emergency presentation occurring within the interval.^18^ From these data, we used linear regression on log (HR) to generate a per-day-delay HR for fatality (delay-HR) (see figure 1).^24 25^ To estimate 10-year survival with no delay under ‘current’ conditions, we applied to NCRAS 10-year survival the COVID-19–related peri-surgical mortality. To estimate 10-year survival encompassing pathway delay, we applied to NCRAS 10-year survival the delay-HR relating to the specified days of delay and also included COVID-19–related peri-surgical mortality.

##### Mortality associated with nosocomial COVID-19 infection

We estimated COVID-associated peri-surgical mortality using per-day estimates for nosocomial infection (2%/5%/10%), operation-specific peri-surgical admission duration and age-specific COVID-19 case fatality rates. Where not under evaluation, the nosocomial infection rate was assumed to be 5% per day of inpatient stay/2.5% for investigatory referral.^26^ We assumed as default that rates of nosocomial COVID-19 infection would halve every 3 months, given improvement in ‘cold’ protocols, personal protective equipment, patient cohorting, and staff and patient testing.

#### Impact of implementing FIT to triage symptomatic patients

We modelled application of FIT to prioritise a subset of symptomatic 2WW CRC referrals out of delay. We modelled the clinically recommended FIT thresholds of 150 µg Hb/g and 10 µg Hb/g, as well as examining the lower threshold of 2 µg Hb/g.^13 16^ We assumed that FIT-positive individuals would go through diagnosis and treatment without delay. We assumed that FIT-negative individuals would receive investigatory referral from primary care for colonoscopy, but that this would be subject to the specified contemporaneous per-patient delay (2/4/6 months). For each FIT threshold, we estimated for each age–stage-specific stratum and in total, the aggregate deaths and lost LYs mitigated by prioritisation along with associated reduction in immediate requirement for colonoscopies.

#### Per-patient risk–benefit analysis for colonoscopy/surgery given nosocomial infection risk

The likelihood of COVID-19 infection from CRC investigatory referral was estimated using nosocomial infection risk of 5% (very high), 2.5% (high) and 1% (moderate). We combined these with the age-specific case-fatality rates^4^ to estimate COVID-related mortality associated with investigatory referral. We also included a ‘technical’ risk of 1 in 10 000 for mortality from perforation and combined the two to produce a combined per-referral mortality.^27^

Based on the 2WW CRC referral-to-diagnosis conversion rates, we estimated at the three rates of nosocomial infection for each age–stage stratum the survival benefit per patient undergoing investigatory referral for colonoscopy. We considered survival advantage from CRC diagnosis versus fatality from procedure (nosocomially acquired COVID-19 or technical) in regard of (1) absolute benefit from investigatory referral and (2) option for referral delay of 2, 4 and 6 months, under the assumption of falling nosocomial infection rates. We performed equivalent risk–benefit analysis at the three rates of nosocomial infection for admission for CRC surgery for diagnosed patients.

All analyses were performed in STATA V.15. The full model and parameter estimates are presented as a dynamic Excel spreadsheet, in which the user can modify key parameters (online supplementary materials).

### Patient and public involvement

This work uses data that have been provided by patients and collected by the NHS as part of their care and support. The data are collated, maintained and quality assured by the National Cancer Registration and Analysis Service, which is part of Public Health England (PHE).

## Results

### Impact of pathway delay on CRC survival per individual

Pathway delays were estimated to be associated with substantial decrements in the 10-year survival of patients with CRC, though the impact varied notably by age, tumour stage and duration of delay. For stage 3 CRC, a 2-month delay to surgery is predicted to cause >9% reduction in survival across all age groups, while for a 6-month delay, this reduction is >29%. Even for stage 1 CRC, the effect of delaying surgery is considerable; a 6-month delay for stage 1 disease is likely to result in a 12.5% reduction in survival for those aged 70–79 (table 1). When considering estimated LYs lost, delays are particularly impactful for younger patients with either stage 2 or stage 3 cancers. For example, for each patient aged 30–39 with stage 3 CRC, 9.4 LYs are lost on average for pathway delay of 4 months, and 15.1 LYs for 6 months of delay (online supplementary table 1).

**Table 1:** Average reduction in 10-year net CRC survival by age and stage consequent from per-patient delay of 2/4/6 months in the diagnostic pathway (assuming no prioritisation based on FIT)

Age band	CRC stage	Average per-patient delay in diagnosis Average reduction in 10-year net survival		
		2 months	4 months	6 months
30–39 years	Stage 1	0.4%	1.1%	1.9%
	Stage 2	5.1%	11.7%	20.1%
	Stage 3	9.1%	20.0%	32.2%
40–49 years	Stage 1	1.6%	3.9%	7.0%
	Stage 2	5.2%	12.0%	20.7%
	Stage 3	9.7%	21.1%	33.5%
50–59 years	Stage 1	1.7%	4.1%	7.5%
	Stage 2	4.9%	11.3%	19.5%
	Stage 3	9.3%	20.5%	32.8%
60–69 years	Stage 1	1.7%	4.3%	8.0%
	Stage 2	5.3%	12.4%	21.4%
	Stage 3	9.5%	20.8%	33.1%
70–79 years	Stage 1	2.7%	6.8%	12.5%
	Stage 2	6.5%	15.0%	25.5%
	Stage 3	11.0%	23.2%	35.0%
80+ years	Stage 1	7.5%	17.2%	28.7%
	Stage 2	8.2%	18.5%	30.4%
	Stage 3	11.5%	22.0%	29.7%

Red shading indicates greater impact on survival; blue shading indicates lesser impact on survival.

### Impact of pathway delay on CRC survival in aggregate across 2WW pathway

On average, 32% (11 229/34 863) of CRC diagnoses come through urgent (2WW) referral. We estimate that an average per-patient delay of 4 months over a single year would result in 1419 deaths and 20 315 lost LYs among patients with CRC diagnosed via this route alone (tables 2 and 3).

**Table 2:** Estimated annual CRC deaths attributable to per-patient average delay in CRC diagnostic pathway of 2/4/6 months (assuming no prioritisation based on FIT)

Age band	CRC stage	Average per-patient delay in diagnosis Aggregate attributable deaths per year in England (2WW CRC cases)		
		2 months	4 months	6 months
30–39 years	Stage 1	0.1	0.2	0.3
	Stage 2	1.3	3.1	5.3
	Stage 3	2.8	6.1	9.8
40–49 years	Stage 1	0.9	2.1	3.8
	Stage 2	5.2	12	20.5
	Stage 3	11.1	24.1	38.3
50–59 years	Stage 1	3.8	9.3	16.9
	Stage 2	20.1	46.6	80.7
	Stage 3	44.5	97.4	155.9
60–69 years	Stage 1	6.4	16	29.6
	Stage 2	36.3	84.7	146.2
	Stage 3	74.9	163.7	260.7
70–79 years	Stage 1	15.3	38.5	70.9
	Stage 2	67.5	156.6	266.3
	Stage 3	133	279.2	421.6
80+ years	Stage 1	36.6	84.4	140.8
	Stage 2	73.5	166.8	273.7
	Stage 3	119.9	228.5	308.9

Based on 10-year net survival and disruption spanning 1 year in which on average 11 226 patients with CRC would be diagnosed with CRC in England via the 2WW pathway. Deeper shading indicates greater impact in attributable deaths.

**Table 3:** Impact of per-patient average delays in CRC diagnostic pathway of 2/4/6 months and impact of mitigation via FIT triage

Reference period of disruption (months)		12		
Duration of background delay (months)		2	4	6
**No FIT prioritisation**	CRC cases	11 226		
	Deaths attributable to delay	653	1419	2250
	Life years lost attributable to delay	9214	20 315	32 799
	Urgent 2WW colonoscopies required	511 394		
**Prioritisation out of delay of individuals with FIT >2 µg Hb/g** Sensitivity: 96.2% Specificity: 64.3%	FIT-positive cases	10 777		
	FIT-negative cases	449		
	Deaths attributable to delay	26	57	90
	Deaths mitigated by FIT prioritisation	627	1363	2160
	Life years lost attributable to delay	369	813	1312
	Lost life years mitigated by FIT prioritisation	8846	19 502	31 487
	Urgent 2WW colonoscopies required	189 216		
**Prioritisation out of delay of individuals with FIT >10 µg Hb/g** Sensitivity: 89.4% Specificity: 83.5%	FIT-positive cases	9991		
	FIT-negative cases	1235		
	Deaths attributable to delay	72	156	248
	Deaths mitigated by FIT prioritisation	581	1263	2003
	Life years lost attributable to delay	1014	2235	3608
	Lost life years mitigated by FIT prioritisation	8201	18 080	29 191
	Urgent 2WW colonoscopies required	92 051		
**Prioritisation out of delay of individuals with FIT >150 µg Hb/g** Sensitivity: 65.9% Specificity: 94.9%	FIT-positive cases	7409		
	FIT-negative cases	3817		
	Deaths attributable to delay	222	482	765
	Deaths mitigated by FIT prioritisation	431	937	1485
	Life years lost attributable to delay	3132	6907	11 152
	Lost life years mitigated by FIT prioritisation	6081	13 408	21 648
	Urgent 2WW colonoscopies required	35 798		

Assumptions: FIT is applied promptly at presentation in primary care and individuals who are FIT-positive are prioritised such that they experience no pathway delay; individuals who are FIT-negative experience the specified ‘background’ pathway delay ahead of being diagnosed.

### Impact of implementing FIT to triage symptomatic patients

Adopting a FIT threshold of 10 µg Hb/g faeces would reduce immediate colonoscopy requirements in the symptomatic group to 18% of normal (92 051/511 394), while still identifying 89% (9991/11 226) of CRCs (table 3). Adopting a higher FIT threshold of 150 µg Hb/g reduces immediate colonoscopy requirements by an additional 56 253 (11%), but at the expense of an additional 150/326/517 deaths per year if background delay rate is 2/4/6 months. A cut-off of 2 µg Hb/g offers improved sensitivity (96%, 10 777/11 226) but only reduces colonoscopies to 37% of normal rates (189 216/511 394).

### Per-patient risk–benefit analysis for colonoscopy/surgery

Addressing concern about COVID-19–related mortality in elderly patients in particular, we considered the risk/benefit trade-offs of investigatory referral and treatment. Even at very high rates of nosocomial infection (5% per investigatory referral), in the symptomatic population investigatory referral versus no investigatory referral offers benefit in cancer survival in every age group. We next addressed the benefit of prompt investigatory referral versus delaying a few months (until risk of nosocomial infection rates could be expected to decrease). Below age 70, prompt colonoscopy offers net survival benefit across all plausible rates of nosocomial infection. However, for those over 70, if the nosocomial risk is high (≥2.5% per procedure), then a delay is preferable if the risk of nosocomial infection is predicted to subsequently decline (table 4).

**Table 4:** Survival benefit from prompt colonoscopy vs delay for different rates of nosocomial infection per investigatory referral

Nosocomial infection rate for investigatory referral	Delay (months)	Age band					
		30–39 years	40–49 years	50–59 years	60–69 years	70–79 years	80+ years
1%	2 months	0.09%	0.09%	0.08%	0.06%	0.04%	0.01%
	4 months	0.21%	0.22%	0.21%	0.20%	0.20%	0.19%
	6 months	0.35%	0.38%	0.36%	0.36%	0.37%	0.36%
2.5%	2 months	0.08%	0.09%	0.06%	0.01%	−0.08%	−0.22%
	4 months	0.20%	0.22%	0.19%	0.14%	0.07%	−0.05%
	6 months	0.35%	0.37%	0.34%	0.30%	0.24%	0.12%
5%	2 months	0.08%	0.08%	0.03%	−0.09%	−0.29%	−0.60%
	4 months	0.20%	0.21%	0.15%	0.04%	−0.14%	−0.44%
	6 months	0.34%	0.36%	0.30%	0.20%	0.02%	−0.28%

This analysis assumes no FIT triage. Green indicates survival benefit from prompt investigatory referral vs delay; red indicates survival disbenefit from prompt investigatory referral vs delay.

Once diagnosed with CRC, comparing prompt surgery against delay for a few months, for nearly all nosocomial infection rates, ages and stages of disease, prompt surgery offered survival advantage (online supplementary table 2).

### Sensitivity analysis

The model was most sensitive to variation in the delay-HR (online supplementary table 1). Varying this by two standard deviations (0.0056±0.009), the total attributable deaths per year for 11 226 CRC cases diagnosed via the 2WW pathway with a uniform per-patient 3-month delay ranged from 838 (delay-HR 0.0047) to 1216 (delay-HR 0.0065). Varying of the per-day rate of nosocomial infection (2%, 5%, 10%) and whether it is static/drops over time had a modest impact on survival estimates.

## Discussion

We have examined using age-specific and stage-specific strata the impact of pathway delays of 2, 4 and 6 months, in terms of overall survival and LYs lost, both by individual and in aggregate, taking into account mortality from nosocomial COVID-19 infection. Predicting that investigatory referral will be the most severe bottleneck in the pathway, we explored the degree of mitigation achieved if 2WW CRC referrals with FIT ≥2, 10 or 150 µg Hb/g were prioritised out of the background delays. Analysing individual-level risk–benefit, we showed net benefit across all ages of investigatory referral without delay, providing the nosocomial infection rate at investigatory referral for endoscopy are moderate or better (≤1%).

### Implications for patient management

Based on linear regression from observational data of diagnosis-to-treatment interval, our analyses suggest CRC pathway delays are associated with substantial impact on survival and LYs lost. Our analyses indicate clear benefit of prompt investigation of patients with symptoms indicative of CRC under most scenarios. If nosocomial infection rates at investigatory referral are high (≥2.5% per referral), for patients aged >70 a short delay may be considered if nosocomial infection rates are likely to decline. Once diagnosed, risk–benefit estimates definitely favour prompt surgery; short delays (2 months) may be favourable only for very early-stage tumours in those aged over 70 in the face of high current per-day nosocomial infection rates (≥5%/day) and only if these nosocomial rates are likely to decline imminently.

### Implications for healthcare planning and resource utilisation

For those patients aged >70 years, in whom most cases of CRC are diagnosed, individual-level risk–benefit trade-off of investigatory referral is predicated on likelihood of nosocomial COVID-19 infection. Rigorous protocols for staff testing and shielding are required, along with an active focus to reserve ‘cold’ sections of the healthcare system for both diagnostics and surgery. This will not only serve to reduce mortality from nosocomial infection, but will improve public confidence in accessing diagnostics and surgery for cancer.

Overall, our analyses strongly suggest that delay in the CRC pathway results in a significant impact on survival with sizeable loss of life years. Tumours will become more advanced, resulting in poorer survival and in the requirement for more costly surgery and/or chemotherapy to manage the upstaged disease. It is also predicted that an appreciable number of patients with CRC initially presenting via the 2WW pathway will, during the period of delay, develop bowel obstruction, perforation or acute bleeding.^28^ The survival impact of these acute events is encompassed in the delay-HR used in our analyses, but our analyses do not reflect the dramatically higher resource requirements for these patients, for example, use of emergency theatre and increased CCU stay.^8 29 30^

Given the dramatic reductions in 2WW referrals during lockdown, it is likely there will be a significant ‘bulge’ in delayed presentations to primary care of symptomatic patients, on top of the normal stream of incident cancer presentations. Given chronic limitations in capacity, stress in the CRC pathway is predicted likely to manifest most prominently at endoscopy.

Improved protocols for protection of endoscopy staff are urgently required to enable support for escalation of colonoscopy from the British Society of Gastroenterology.^11 31^ FIT-based prioritisation offers the opportunity for short-term rationalisation during times of extreme restriction to endoscopy services. However, sensitivity of FIT for CRC at thresholds of 10–150 µg Hb/g translate in a symptomatic population to negative predictive values that are unacceptable; hence, FIT-negative symptomatic patients will still require colonoscopy. Furthermore, use of currently recommended FIT thresholds of 150 µg Hb/g will miss 33% of CRCs and also many high-risk adenomas too. Triage algorithms in symptomatic patients would be much improved by incorporation of specific symptomatology, gender and risk factors such as obesity and family history.^32^ While use of CT/colonography has been expanded in some areas, we have not further examined these approaches as (1) UK professional groups are also currently advising against colonography on account of infection risk, (2) negative predictive value is poor compared with colonoscopy, (3) diagnostic tissue cannot be obtained, and (4) radiology services are also under significant pressure.

Unless supra-normal capacity is made available rapidly within endoscopy/diagnostics and surgery, knock-on delays may persist for months or even years. Exacerbating this, throughput in outpatients, radiology, endoscopy and theatres is likely to be slower, on account of infection control redesign required to include social distancing and employment of personal protective equipment. Beyond recovery from this first ‘bulge’, we should pay urgent attention to building sufficient capacity in pathways for CRC diagnostics and treatment, to provide resilience against disruption from subsequent waves of COVID infection or other health crises which compromise clinical resources.

### International relevance

While we have used data specifically for England, CRC diagnosis, management and survival are directly comparable across most economically developed countries, so the impact of delay that we highlight is broadly applicable. However, our model focuses on elective diagnosis in patients with symptoms suggestive of CRC as defined by the UK criteria for urgent (2WW) referral; there will be variation internationally by eligibility and patient distribution within pathways to CRC diagnosis for asymptomatic/symptomatic patients.

### Limitations of analysis

As with any model-based analysis, the accuracy of our predictions is dependent on the validity of assumptions and estimates used for parameterisation. As per the sensitivity analysis and detailed in our previous health-systems analyses of cancer pathway delays, the outputs of our model are predicated heavily on accuracy of the delay-HR.^25^ In studies of screening programmes for asymptomatic patients, longer delays to colonoscopic investigation have been associated with poorer CRC outcomes.^33 34^ In studies of symptomatic patients, there is a highly heterogeneous literature with a widely reported ‘U-shaped’ relationship between survival and diagnostic delay, which has been ascribed to so-called ‘confounding by indication’ (‘waiting time paradox’ or ‘sicker-quicker’ phenomenon).^35–38^ Accordingly, weak and even negative associations between delay and outcome from studies focused on shorter windows have been reported but likely just reflect the prioritisation of sicker patients.^38^ In order to consider long-term survival, we required partitioning of stage 1–3 cancers potentially curable by surgery from stage 4 cancers in which delays would be predicted to have minimal impact on long-term survival. We, therefore, derived our delay-HR estimates from Taiwanese CRC registry data, as this was the only study identified in which long-term outcome was correlated with diagnosis-to-treatment interval stratified by stage; furthermore, it was by far the largest study identified (>39 000 CRC diagnoses) and captured sizeable numbers with longer periods of delay (2533 (6.4%) had delay of 31–150 days and 1252 (3.15%) had delay of >151 days).^18^ Although these data do not support significant stage-specific heterogeneity in the effect of delay in stages 1–3, we present an option for the reader to explore the impact of potential heterogeneity on model outputs, in combination with different per-patient delays, duration of disruption and nosocomial infection rates (online supplementary materials).

These are non-naturalistic ‘what-if’ analyses in which we explore impact of a universal per-patient pathway delay lasting 1 year: in reality, delays will vary temporally, geographically and individually and the overall duration of disruption has yet to play out. There may be serial delays at different stages in the patient pathway from first symptoms to completion of treatment; these bottlenecks may be complex and interdependent. Our approach is explicitly survival focused: a more elaborate model capturing stage transition may offer additional utility for healthcare planning. While most 2WW referrals are typically investigated using colonoscopy, data are not available to specify the proportion typically diverted in secondary care to alternative investigation such as CT/colonography. Under normal circumstances, 0.6% of 2WW colorectal referrals lead to the eventual diagnosis of another cancer type^10^: we have not been able to capture the impact of delay on diagnoses of these cancers.

We have focused exclusively on pathways for ‘urgent’ symptomatic patients and have not considered the other routes to diagnosis (screening, routine, emergency). Sustained contraction of routine endoscopy services may result in the diversion to the 2WW pathway of CRC diagnoses that otherwise would have been made in asymptomatic patients via screening or routine endoscopic follow-up^28^; potential alterations to 2WW volumes by these groups have not been taken into account.

We have focused exclusively on the detection of invasive CRC. Due to lack of data on the frequency and distribution of pre-invasive adenomas or robust observation data by which to model progression, we have not included in our analyses the impact of delayed detection and removal of non-invasive adenomas. Hence, our estimates of the impact of delays will be inherently conservative.

We have not evaluated alterations in systemic anti-cancer therapy (SACT) delivery, bearing in mind that SACTs make comparatively limited contribution to CRC survival, in particular for rectal cancer. As we have focused on long-term survival afforded from surgery with curative intent, we have not modelled impact of delay on outcomes for stage 4 cancers, instead, making the assumption that treatment delay has no impact on long-term survival for this group.

## Conclusion

We estimate that even short delays in the pathway to CRC treatment will result in significant mortality and lost LYs. Provided nosocomial infection rates are controlled, there is net survival benefit in all age groups for prompt investigatory referral for colonoscopy. FIT-based prioritisation could be of utility short term to prioritise FIT-positive symptomatic patients out of delay and reduce pressure on endoscopy services. Given that disruption of cancer services may persist for multiple years due to successive waves of the COVID-19 pandemic, in order to avoid substantial numbers of deaths attributable to pathway delay, urgent attention is required to minimise and mitigate disruption to CRC diagnostics and treatment.

## Data Availability

All data relevant to the study are included in the article or uploaded as online supplementary information.
