# Supplementary material for: Prioritisation by FIT to mitigate the impact of delays in the 2-week wait colorectal cancer referral pathway during the COVID-19 pandemic: a UK modelling study
Source: Gut. 2020 Aug 27;70(6):1053–60. doi: 10.1136/gutjnl-2020-321650 (PMC7447105; doi:10.1136/gutjnl-2020-321650)
Supplement: Supplementary data [file gutjnl-2020-321650supp002.pdf]

Prioritisation by FIT to mitigate the impact of delays in the two-week wait colorectal cancer referral pathway during the COVID-19 pandemic: a UK modelling study

Chey Loveday[1*], Amit Sud[1*], Michael Jones[1*], John Broggio[2], Stephen Scott[3], Firza Gronthoud[4], Bethany Torr[1], Alice Garrett[1], David L. Nicol[5,6], Shaman Jhanji[7,8], Stephen A. Boyce[9], Matthew Williams[10,11] , Claire Barry [3], Elio Riboli[12], Emma Kipps[3,13], Ethna McFerran[14], David C. Muller[13,], Georgios Lyratzopoulos[2,15], Mark Lawler[14], Muti Abulafi[16], Richard Houlston[1,17], Clare Turnbull[1,2,17]

*these authors contributed equally to the work

1. Division of Genetics and Epidemiology, Institute of Cancer Research, London, UK.
2. National Cancer Registration and Analysis Service, Public Health England, Wellington House, London, UK.
3. RM Partners, West London Cancer Alliance, London, UK.
4. Microbiology, Royal Marsden NHS Foundation Trust, London, United Kingdom
5. Urology Unit, Royal Marsden NHS Foundation Trust, London, UK.
6. Division of Clinical Studies, Institute of Cancer Research, London, UK.
7. Department of Anaesthesia, Perioperative Medicine and Critical Care, Royal Marsden NHS Foundation Trust, London, UK
8. Division of Cancer Biology, Institute of Cancer Research, London, UK
9. Department of Colorectal Surgery, Oxford University Hospitals NHS Foundation Trust, Oxford, UK.
10. Department of Clinical Oncology, Imperial College Healthcare NHS Trust, London, UK.
11. Computational Oncology Group, Imperial College London, London, UK.
12. School of Public Health, Imperial College London, UK.
13. The Breast Unit, Royal Marsden NHS Foundation Trust, London, UK.
14. Patrick G Johnston Centre for Cancer Research, School of Medicine, Dentistry and Biomedical Sciences, Queen's University Belfast, Belfast, UK.
15. Epidemiology of Cancer Healthcare and Outcomes (ECHO) Group, University College London, London, UK.
16. Colorectal Surgery, Croydon Health Services NHS Trust, on behalf of RMP NICE FIT Steering Group, London, UK.
17. Department of Clinical Genetics, Royal Marsden NHS Foundation Trust, London, UK.

Corresponding author: Clare Turnbull; address: Division of Genetics and Epidemiology, Institute of Cancer Research, London, UK, SM2 5NG; email: Clare.Turnbull@icr.ac.uk; tel: 020 8722 4487.

Loveday C, *et al. Gut* 2021; 70:1053–1060. doi: 10.1136/gutjnl-2020-321650

Reference period of disruption (months)		12								
Duration of background delay (months)		3								
Pan-stage per-day delay-HR		0.0056			0.0065 (+2SD)			0.0047 (-2SD)		
Per-day nosocomial infection rate halves each 3 months	Nosocomial infection rate (per-day): current	2.0%	5.0%	10.0%	2.0%	5.0%	10.0%	2.0%	5.0%	10.0%
	Nosocomial infection rate (per-day): post-d	1.0%	2.5%	5.0%	1.0%	2.5%	5.0%	1.0%	2.5%	5.0%
	Attributable deaths	**1,062**	**1,025**	**978**	**1,255**	**1,216**	**1,168**	**874**	**838**	**793**
	Life years lost	**14,949**	**14,552**	**14,064**	**17,730**	**17,320**	**16,812**	**12,256**	**11,872**	**11,403**
Per-day nosocomial infection rate is stable	Nosocomial infection rate (per-day): current	2.0%	5.0%	10.0%	2.0%	5.0%	10.0%	2.0%	5.0%	10.0%
	Nosocomial infection rate (per-day): post-d	2.0%	5.0%	10.0%	2.0%	5.0%	10.0%	2.0%	5.0%	10.0%
	Attributable deaths	**1,081**	**1,067**	**1,047**	**1,273**	**1,256**	**1,233**	**894**	**882**	**866**
	Life years lost	**15,161**	**15,025**	**14,840**	**17,934**	**17,773**	**17,556**	**12,477**	**12,364**	**12,211**

Supplementary Table 1: Multivariate sensitivity analysis
Hilighted in yellow is the estimated impact of 3 months delay to treatment under default parameter assumptions (Per-day delay-HR=0.0056, per-day nosocomial infection rate=5%, nosocomial infection rate halves each 3 months)

Loveday C, *et al. Gut* 2021; 70:1053–1060. doi: 10.1136/gutjnl-2020-321650

**Age band**	**Stage**	**Average per-patient delay in diagnosis** Average reduction in life years		
		**2 months**	**4 months**	**6 months**
30-39 years	Stage 1	0.2	0.5	0.9
	Stage 2	2.4	5.5	9.5
	Stage 3	4.3	9.4	15.1
40-49 years	Stage 1	0.6	1.5	2.6
	Stage 2	2	4.5	7.8
	Stage 3	3.6	7.9	12.5
50-59 years	Stage 1	0.5	1.2	2.1
	Stage 2	1.4	3.2	5.5
	Stage 3	2.6	5.8	9.3
60-69 years	Stage 1	0.3	0.9	1.6
	Stage 2	1.1	2.5	4.2
	Stage 3	1.9	4.1	6.6
70-79 years	Stage 1	0.3	0.8	1.5
	Stage 2	0.8	1.8	3.1
	Stage 3	1.4	2.8	4.3
80+ years	Stage 1	0.5	1.1	1.8
	Stage 2	0.5	1.2	1.9
	Stage 3	0.7	1.4	1.9

Supplementary Table 1: Reduction in life years from delay in colorectal cancer treatment (based on 10-year net survival). The strength of shade of orange indicates the greatest loss in life years from delay.

Loveday C, *et al. Gut* 2021; 70:1053–1060. doi: 10.1136/gutjnl-2020-321650

		2%			5%			10%		
		**2 months**	**4 months**	**6 months**	**2 months**	**4 months**	**6 months**	**2 months**	**4 months**	**6 months**
30-39 years	Stage 1	0.4%	1.1%	1.9%	0.4%	1.0%	1.9%	0.3%	1.0%	1.8%
	Stage 2	5.1%	11.7%	20.1%	5.0%	11.7%	20.1%	5.0%	11.6%	20.0%
	Stage 3	9.1%	20.0%	32.2%	9.0%	19.9%	32.1%	9.0%	19.9%	32.0%
40-49 years	Stage 1	1.6%	3.9%	7.0%	1.5%	3.8%	6.9%	1.4%	3.7%	6.8%
	Stage 2	5.2%	12.0%	20.7%	5.1%	11.9%	20.6%	5.0%	11.8%	20.5%
	Stage 3	9.7%	21.0%	33.5%	9.6%	21.0%	33.4%	9.5%	20.8%	33.2%
50-59 years	Stage 1	1.6%	4.1%	7.5%	1.4%	3.8%	7.2%	1.0%	3.4%	6.7%
	Stage 2	4.8%	11.3%	19.5%	4.5%	11.0%	19.2%	4.2%	10.6%	18.8%
	Stage 3	9.3%	20.4%	32.7%	9.0%	20.1%	32.4%	8.7%	19.7%	32.0%
60-69 years	Stage 1	1.5%	4.2%	7.9%	0.8%	3.4%	7.0%	-0.2%	2.3%	5.8%
	Stage 2	5.1%	12.3%	21.4%	4.4%	11.5%	20.5%	3.4%	10.4%	19.3%
	Stage 3	9.3%	20.7%	33.1%	8.6%	19.9%	32.2%	7.6%	18.8%	31.0%
70-79 years	Stage 1	2.2%	6.5%	12.4%	0.6%	4.6%	10.3%	-1.5%	2.2%	7.7%
	Stage 2	5.9%	14.7%	25.4%	4.3%	12.9%	23.4%	2.3%	10.5%	20.7%
	Stage 3	10.5%	22.8%	34.7%	8.9%	21.1%	32.9%	6.9%	18.8%	30.5%
80+ years	Stage 1	6.4%	16.6%	28.3%	3.5%	13.3%	24.7%	-0.3%	8.8%	19.9%
	Stage 2	7.1%	17.9%	30.0%	4.2%	14.6%	26.4%	0.4%	10.2%	21.7%
	Stage 3	10.3%	21.0%	28.8%	7.6%	18.1%	25.8%	4.0%	14.1%	21.7%

Supplementary Table 2: Survival benefit from surgery versus delay, for different per day rates of COVID-19 nosocomial infection (assuming 6 day in-patient stay and rates of nosocomial infection halve for every 3 months of delay). Green colour indicates survival benefit from avoidance of delay; red colouring indicates survival benefit from delay.

Loveday C, *et al. Gut* 2021; 70:1053–1060. doi: 10.1136/gutjnl-2020-321650
